# Leaky Ryanodine receptors increases Amyloid-beta load and induces memory impairments in Tg2576 mouse model of Alzheimer disease

**DOI:** 10.1186/1750-1326-8-S1-P54

**Published:** 2013-10-04

**Authors:** Bénédicte Oulès, Dolores Del Prete, Barbara Greco, Xuexin Zhang, Inger Lauritzen, Mohamed Trebak, Fabio Benfenati, Frédéric Checler, Mounia Chami

**Affiliations:** 1 INSERM U 807, Paris V University, F-75015 Paris, France; 2 Neuroscience and Brain Technologies - Istituto Italiano di Tecnologia, 16163 Genova, Italy; 3 Institut de Pharmacologie Moléculaire et Cellulaire, UMR7275 CNRS/UNSA, 06560 Valbonne, France; 4 Center for Cardiovascular Sciences, MC8, Albany Medical College, Albany, NY 12208; USA

## Background

In Alzheimer disease (AD), the perturbation of the endoplasmic reticulum (ER) calcium (Ca2+) homeostasis has been linked to presenilins (PS) [1], the catalytic core in gamma-secretase complexes cleaving the amyloid precursor protein (APP) thereby generating amyloid beta peptides. Here we investigate whether APP perturbs ER Ca2+ homeostasis and whether ER Ca2+ could in turn influence amyloid beta production.

## Materials and methods

We used neuroblastoma SH-SY5Y cell lines stably overexpressing human APP695 cDNA harboring or not the Swedish double mutation (K670M→N671L) (APPswe and APP695 respectively) and Tg2576 mice carrying human APPswe cDNA under the control of the hamster Prion promoter. We analyzed subcellular Ca2+ signals by using aequorin targeted Ca2+ probes, Fura2, AM dye and patch clamp experiments. We analyzed the expression of the Ryanodine receptor (RyR) by western blot and quantitative RT-PCR. We studied the impact of RyR blockade by dantrolene on APP metabolism and Amyloid beta peptide production using biochemistry, ELISA and immunohistochemistry approaches and investigated the underlying mechanisms through the study of APP phosphorylation, and beta- and gamma secretase expression and activities. We finally studied pre- and post-synaptic proteins expression and learning and memory paradigms in Tg2576 mice upon vehicle or dantrolene treatment.

## Results

We show that overexpression of APP695, or APPswe triggers increased Ryanodine receptors (RyR) expression and enhances RyR-mediated ER Ca2+ release in SH-SY5Y neuroblastoma cells and in APPswe-expressing (Tg2576) mice. Interestingly, dantrolene-induced lowering of RyR-mediated Ca2+ release leads to the reduction of both intracellular and extracellular amyloid beta load in neuroblastoma cells as well as in primary cultured neurons derived from Tg2576 mice. This Aβ reduction can be accounted for by decreased Thr-668-dependent APP phosphorylation and β- and gammaological lesions and slows down learning and memory deficits in Tg2576 mice [2].

## Conclusions

Overall, our data document a key role of RyR in Aβ production and learning and memory performances, and delineate RyR-mediated control of Ca2+ homeostasis as a physiological paradigm that could be targeted for innovative therapeutic approaches.
